# ASSOCIATION BETWEEN MULTIMORBIDITY AND KIDNEY FUNCTION DECLINE IN OLD AGE: A SWEDISH POPULATION-BASED COHORT STUDY

**DOI:** 10.1093/geroni/igae098.2396

**Published:** 2024-12-31

**Authors:** Giorgi Beridze, Lu Dai, Juan-Jesús Carrero, Alessandra Marengoni, Davide Liborio Vetrano, Amaia Calderón-Larrañaga

**Affiliations:** Karolinska Institutet, Stockholm, Stockholms Lan, Sweden; Karolinska Institutet, Stockholm, Stockholms Lan, Sweden; Karolinska Institutet, Stockholm, Stockholms Lan, Sweden; University of Brescia, Brescia, Lombardia, Italy; Karolinska Institutet, Stockholm, Stockholms Lan, Sweden; Karolinska Institutet, Stockholm, Stockholms Lan, Sweden

## Abstract

We aimed to study the associations between multimorbidity (the presence of ≥2 conditions) and multimorbidity patterns, and the risk of kidney function decline in older adults. 3094 individuals from the Swedish National Study on Aging and Care in Kungsholmen (SNAC-K) were followed between 2001 and 2016. Multimorbidity was operationalised as the number of chronic conditions and multimorbidity patterns identified using latent class analysis. Joint models and Cox regression models were used to explore the associations between multimorbidity, and absolute and relative (≥25% decline from baseline) changes, respectively, in the estimated glomerular filtration rate (eGFR) calculated using the creatinine-based Berlin Initiative Study equation. Mean age of the sample was 73.9 and 87% had multimorbidity. There was a dose-response relationship between the number of chronic conditions, and absolute (β [95% CI] = -0.05 [-0.07;-0.03]) and relative (HR [95% CI] = 1.23 [1.17;1.29]) declines in eGFR. Five patterns of multimorbidity were identified. The Unspecific, low burden pattern had the lowest morbidity burden and was used as the reference category. The Unspecific, high burden and Cardiometabolic patterns showed accelerated absolute (β [95% CI] = -0.15 [-0.26;-0.05] and -0.77 [-0.98;-0.55], respectively) and relative (HR [95% CI] = 1.45 [1.09;1.92] and 3.45 [2.27;5.23], respectively) declines. Additionally, the Cognitive and Sensory pattern showed accelerated relative decline (HR [95% CI] = 1.53 [1.02;2.31]). No associations were found for the Psychiatric and Respiratory pattern. Multimorbidity is strongly associated with accelerated kidney function decline in older age. Individuals with cardiometabolic multimorbidity exhibit a particularly increased risk.

